# Rethinking gamete donor care: A satisfaction survey of egg and sperm donors in the UK

**DOI:** 10.1371/journal.pone.0199971

**Published:** 2018-07-23

**Authors:** Richard A. Williams, Laura L. Machin

**Affiliations:** 1 Department of Management Science, Management School, Lancaster University, Lancaster, United Kingdom; 2 Data Science Institute, Lancaster University, Lancaster, United Kingdom; 3 Medical School, Faculty of Health and Medicine, Lancaster University, Lancaster, United Kingdom; Cornell University College of Veterinary Medicine, UNITED STATES

## Abstract

**Objective:**

Despite poor clinic communication and staff treatment being reported by donors, high rates of overall satisfaction are still reported in surveys. This study will evaluate the importance of communication and interaction between donors and fertility clinic staff in gamete donor care.

**Methods:**

We report on 120 egg and sperm donors’ responses to a UK-wide online satisfaction survey. The survey focused on donors’ interactions with fertility clinic staff pre-, during, and post- donation. Basic cross-tabulation was performed on the data using online survey software. Textual data was read and extracts identified, which illustrated and expanded on the findings from the numerical data. Diagrammatic modeling was also utilised to analyse the textual data, with particular focus to relationships between the donors and clinic staff, the main activities within the gamete donation process, and how these activities may affect donor satisfaction with the gamete donation process.

**Results:**

Donors expressed concern for the infertile couple and the resulting child; conveyed frustration at not receiving information on the expenses they could claim; felt lost in the system regarding the ease of making clinic appointments, and once made they were routinely not seen on time for these appointments. Donors also negatively commented on aftercare, the location and condition of the donation room, and information on contraception. In addition, Ovarian Hyperstimulation Syndrome was frequently reported, with these egg donors believing that clinic staff were not concerned with their physical or emotional well-being, but were instead disproportionately focused on extracting the eggs.

**Conclusions:**

The multifaceted notion of donors highlights the complexity inherent to the gamete donation process, which comprises various aspects of uncertainty in the donation system, and ambiguity in the donation process. Categorising donors as Altruist, Customer, and Patient, conveys the particular importance of staff communication and treatment in donor care. These categories are not mutually exclusive however, in that an individual donor may experience more than one of these perspectives during the course of their gamete donation journey. Finally, there were a number of exemplar cases, where donors reported high satisfaction throughout, and these correlated with them being given a single point of contact at the clinic. Subject to resource constraints, we suggest that this practice should be implemented throughout clinics in the UK, so that donors have access to dedicated clinic staff who not only support them emotionally and physically throughout the gamete donation process, but also ensure that communication is open, clear, timely, and consistent.

## Introduction

The communication and interaction between gamete donors and fertility clinic staff have frequently been explored through satisfaction surveys, and have highlighted aspects of the donation process that donors perceive in need of improvement. Previous studies have found that donors feel disrespected and treated as a commodity by clinic staff [1]. Yet, despite poor clinic communication and staff treatment being reported by donors, and identified as key areas for improvement, high rates of overall satisfaction are still reported in surveys [2, 3]. For some, the method of accessing the donors’ views has been identified as accounting for this phenomenon [4]. However, a closer examination of satisfaction surveys, suggests that the expressions of satisfaction may reflect respondents’ perceptions of staff, such as they are performing under difficult circumstances [5], or what is being requested of them is beyond their role [6]. For example, Allan [7] highlighted how fertility patients acknowledged the structural and temporal restrictions on fertility nurses to provide ‘good enough’ emotional care when attending clinics. In essence, it cannot be assumed that positive overall satisfaction survey results mean respondents’ expectations on *all* aspects of the donation process have been fulfilled [8, 9], but instead are accepted and tolerated.

In this paper, we consider this alternative interpretation for high overall satisfaction ratings, despite the poor communication and staff treatment reported by donors, through analysing 120 egg and sperm donors’ responses in a UK-wide satisfaction study. The contributions of communication (from clinic staff to donors) and interaction (two-way between clinic staff and donors) in providing gamete donor care are explored, which allow for recommendations to be made regarding the interaction between donors and clinic staff, with particular reference to fertility nurses. Such recommendations are timely, given an increase in donations is required in order for gamete donation to continue as a viable method of conception [10, 11].

### Background

High overall satisfaction ratings may account for why the matter of improving gamete donor care has tended to be overlooked and only recently reached the policy agenda. In 2012, the Human Fertilisation and Embryology Authority (HFEA) formed the National Donation Strategy Group to assist with *‘…improving the customer service that donors receive when they contact clinics…’* [12]. Furthermore, calls for dedicated clinic staff to support gamete donors throughout the various stages of the donation process have increased [13]. Fertility nurses are ideally placed to respond to this call given the guidance and advice they already provide to fertility patients, which is known to influence patients’ choices and motivation [4]. Fertility nurses’ contact with patients tends to be throughout the treatment process [14] thereby enabling patients to feel closer to, and more able to communicate with nurses, compared to other professionals [15]. In turn, fertility nurses have been found to appreciate the increased personal contact with patients when new patient initiatives have been introduced in fertility clinics [16].

In 2017, the matter of egg donor care was highlighted in the media, as clinics were alleged to have underreported cases of Ovarian Hyperstimulation Syndrome (OHSS) to the HFEA [17]. OHSS is a side effect of the fertility drugs used to stimulate the ovaries to release eggs, with symptoms including abdominal swelling, nausea, dehydration, and difficulty breathing [18]. Figures available from the HFEA only refer to *serious* or *critical* OHSS, with 60 cases reported in 2015 and 38 in 2016; clinics are not required to report *mild* or *moderate* cases of OHSS to the HFEA, meaning it is hard to get a sense of the full scale of the problem [18].

A consequence of this focus on gamete donor care is that the framing of donors is evolving. Donors are being constructed as customers in terms of the ‘service’ they require from clinics [19], which stands in sharp contrast to the long-standing constructions of donors as altruistic based on the descriptions of: the side effects, pain, and inconvenience donors endure; their refusal of financial payment in return for their donation; and their desire for knowledge of donation outcomes; or disappointment when their donation does not result in pregnancy [20–22]. Men and women are increasingly being discussed as ‘consumers’ rather than ‘patients’ [9], and concerns have been raised as to how such a shift in terminology can mask (negative) aspects of the healthcare experience for the ‘consumer-patients’ [5]. Furthermore, until recently, distinctions have routinely been made between people donating their eggs or sperm to be used in fertility treatment, against those attending the clinic for fertility treament [19, 23], or those requiring medical treatment [24]. However, satisfaction studies with the donation process in Sweden have called for clinic staff to view donors as patients in order to take into account their attitudes and experiences, thereby improving the care they receive [25, 26].

Caring is considered central to the role of nursing, with fertility patients valuing the practical, everyday skills that fertility nurses used to care for them [27]. Researchers have therefore called for recognition of fertility nurses’ role and how it can contribute to the positive experience of those undergoing fertility treatment [14]. This study builds on these findings to demonstrate how fertility nurses can also play a postive role for those attending clinics to donate gametes.

## Methods

### Aims

The National Gamete Donation Trust (NGDT) is a national body within the United Kingdom (UK), which works to raise the awareness of gamete donation. In 2010, they began a multi-year project that set out to explore how satisfied, egg and sperm donors were, with the donation process in the UK and how it could be improved.

### Research design

A questionnaire was designed for egg donors and sperm donors, by senior members of the NGDT to reflect these aims. A member of the research team (LLM) had oversight of the questionnaire, and provided guidance on phrasing and positioning of questions asked, along with the format and content of the questionnaire. Each questionnaire included closed and open questions, thereby generating quantitative and qualitative data. Where relevant, the same questions were asked of egg donors and sperm donors.

The focus of the questionnaires was the donation process and respondents’ contact with the clinics, including the aftercare offered by the clinics, and respondents’ experiences of counselling. To reflect current policy and practice interests, the questionnaires asked about donors’ access to a single point of contact at clinics, staff assistance with writing Goodwill Messages and Personal Descriptions for any child conceived through donation, and donors receiving expenses. Each questionnaire concluded with respondents being asked to rate their donation experience, and whether they would consider donating again in the future.

The questionnaires were designed to avoid collecting an excessive amount of demographic data, such as employment status or age. However, this could be considered a limitation of the design of the questionnaires as others [28] have collected this data, since it is possible that respondents’ employment status may have influenced their responses relating to expenses for donors.

### Study sample

The aim was to recruit men and women who had donated their eggs or sperm at a UK clinic. However, this population of respondents are difficult to identify, because accurate records that detail every person who has donated at a UK clinic are not accessible from the HFEA. As a result, it was not possible to determine a ratio of sample size to population. It also made recruiting donors challenging. Consequently, the eligibility criteria to participate in the study were broad. No time restrictions were given as to when the donation had taken place, nor were donors from specific geographic regions targeted, or at particular clinics. It is important to remember that the circumstances surrounding each respondents’ donation may differ according to national ploicy and practice guidelines. Significantly, for this study, when interpreting the data, the HFEA held a public consultation in 2011 on gamete donation, which resulted in a shift away from out of pocket expenses and a loss of earnings allowance (previously capped at £250), to sperm donors receiving a fixed sum of £35 per clinic visit, and egg donors receiving a fixed sum of £750 per cycle from April 2012 [29]. Furthermore, donor anonymity was removed in 2005, meaning that adults who are aware of their donor-conception origins, have the option to access identifying information about their donors. However, it is not possible for donors to contact adults conceived through their gamtetes, although they do have the option to write a Good Will Message for anyone conceived through their gametes (can be accessed once the child reaches adulthood). At the time of writing, the policies surrounding donation, such as expenses and anonymity are still in place, and our findings are relevant to practice.

### Data collection

Recruitment took place between May 2011 and August 2012. Adverts were placed on the NGDT website, and were sent to NGDT members via email. The study was also promoted to donor co-ordinators and fertility counsellors. The questionnaire was accessed through the NGDT website and completed online.

To encourage ‘true’ respondents completed the questionnaire, no (financial) incentives were offered to promote participation. In addition, completing the questionnaire was lengthy and time consuming, therefore making it an unappealling activity for those who did not meet the eligibility criteria.

Respondents were asked to complete the questionnaire with a single donation experience in mind. However, it is possible that a respondent could have answered the same question differently depending on the various occasions they donated. Respondents were not asked to justify which donation ‘experience’ they referred to when completing the questionnaire. Furthermore, respondents were self-selecting. As a result, the study may have attracted respondents who had significantly negative or positive experiences [23] and were therefore more inclined to take part. However, the study findings do not support this, as donors’ experiences were found to be complex, for example, an individual respondent reported experiencing one aspect of the donation process positively and another aspect of the process negatively.

### Response rate

A total of 120 people completed the questionnaires. Seventy two respondents were egg donors and 48 respondents were sperm donors (n = 120). A response rate to this study cannot be determined as it is not possible to state how many donors chose not to participate after reading the study advert. Furthermore, accurate records that detail every person who has donated at a UK clinic are not accessible via the HFEA, resulting in an inability to calculate in statistical terms how representative the sample is of the wider UK donor population.

All respondents completed over half of their questionnaire. As no question was deemed a priority in order to interpret the data, all questionnaires have been included in the total response rate. However, to be as accurate as possible in reporting the data, the total number of respondents who answered a specific question is provided.

### Data analysis

Basic cross-tabulation was performed on the data using online survey software, allowing for the data to be summarised in this paper. Pairwise comparison was initially performed, leading up to more complex combinations, for example egg and sperm donors who rate their overall experience positively and had counselling, in order to identify if, and what, relationships exist between them [30]. Three approaches were adopted to the cross-tabulation of the data. Each approach was applied separately to the data set, and analytically significant observations were noted. Firstly, all respondents’ answers to the same question were compared, where possible, to explore if one group of respondents experienced elements of the donation process differently to the other group. For example, did more egg donors rate their interaction with clinics positively compared to sperm donors, or did sperm donors rate their experiences of aftercare higher than egg donors. Secondly, all respondents within a specific group, e.g. egg or sperm, were grouped according to specific categories, such as whether they defined themselves as ‘sharers’ or ‘donors’, whether they had donated to strangers or known recipients, or whether they had donated before or after the removal of donor anonymity in the UK in 2005. Again, this was to examine if the donation process was reported differently according to the personal and donor characteristics of the respondent. Answers across all respondents were also grouped according to clinics they attended to see if there were any similarities. However, the imbalance in the number of respondents across the demographic categories, i.e. more donors than sharers, meant this approach to analyse the data was curtailed. As a result, the term ‘donor’ in this paper incoporates the experiences of those respondents who defined themselves as ‘sharers’, as well as ‘donors’. Thirdly, an individual respondent’s questionnaire was assessed if he or she gave answers that were at the extremes of the set of responses to a question, i.e. ‘very good’ or ‘very poor’. This suggested that the respondent had strong feelings surrounding [a] specific aspect(s) of the donation process and therefore could generate analytically interesting findings. Conducting the analysis in this way ensures that the entire data set was interrogated comprehensively.

The qualitative data were read and coded according to themes. Codes initially derived from the research questions reflected very broad themes, such as the ‘portrayal of donors’ and ‘portrayal of staff’. On average, the qualitative data on each questionnaire was read at least three times, with new codes emerging with each reading or existing codes becoming more refined. Importantly, any ‘unexpected issues’ [31] that emerged during the reading of the data were also acknowledged, which resulted in further refinement of the codes. Finally, the codes were considered in light of the research questions and were brought together to illustrate the analysis [32].

### Diagrammatic modelling

Perhaps the simplest approach to modelling the interactions between components/actors within a complex system (biological, physical, and social), and the resultant emergent behaviours of the system following these interactions, are diagrammatic in nature. A number of approaches have been developed since the 1990s, which have borrowed standardised diagrammatic languages from computer science. One such standard is the Unified Modelling Language (UML) [33] and [34], which although originally developed to document technical requirements for the analysis and design of computer systems [35], has recently been used to model complex biological systems.

The diagrammatical modeling was performed in an iterative manner following completion of the thematic coding of the qualitative data. We have chosen to follow the approach of [36] (who used UML to model fertility treatment and gamete donation) and [37] (who used UML to model a complex system from relational biology), and [38] (who used UML to model an aspect of the immune system), in using UML as the basis to semi-formally define the interactions between donors and clinic staff (UML Communication Diagram), and the main activities involved in the gamete donation process (UML Activity Diagram). Along with the two diagrammatic UML notations (UML v2.4 [39]), a less formal cartoon diagram was also used to ensure the complexity inherent to the donor’s perception of their overall satisfaction with the gamete donation process could be conveyed efficiently. This diagrammatic notation has been deemed an *Emergent Behaviours Diagram* (after [40], who diagrammatically modelled a signalling pathway involved in the human immune system), which was itself based upon the *Rich Picture* diagrammatic notation used in the wider discipline of Management Science (specifically, the Soft Systems Methodology approach, after [41]).

### Ethics approval

An application for ethics approval was reviewed by Lancaster University Research Ethics Committee, and approved by the committee on 11^th^ December 2011. Before accessing the online questionnaire, respondents were directed to a webpage that detailed information on who would have access to the data, how it would be used, and for what purposes it was being collected, to ensure informed consent was gathered. Limited identifiable data were collected from respondents, such as names or addresses. A respondent code was allocated, such as ‘ED_02’ to represent the respondent as an egg donor and was the second respondent to complete the questionnaire.

## Results

### Demographics

S1 Table represents the data from the questionnaire responses that has been used for the statistical analysis in this manuscript. Table 1 summarises the combined demographic responses from male and female respondents. Over half of all respondents had their own children when they made their donation. The male respondents were more likely to be single at the time of their donation than the female respondents. Table 2 summarises the respondents’ donation profile. Over three quarters of all respondents had donated to someone they did not know. The male respondents had donated on more separate occasions, compared with female respondents. Over three quarters of all respondents were aware of how many families had received their donation.

**Table 1:** Respondents’ demographics. Tabulated summary of respondents’ demographics at the time of their donation.

	Female Respondents	Male Respondents	All Respondents
I have donated my gametes to be used for another person’s fertility treatment	56 (n = 69) (81%)	44 (n = 45) (98%)	100 (n = 114) (88%)
I have shared my gametes for another person’s fertility treatment	12 (n = 69) (17%)	0 (n = 45) (0%)	12 (n = 114) (11%)
I have both donated and shared my gametes for another person’s fertility treatment	1 (n = 69) (1%)	1 (n = 45) (2%)	2 (n = 114) (2%)
I donated before the removal of donor anonymity in the UK	2 (n = 67) (3%)	2 (n = 45) (4%)	4 (n = 112) (4%)
I had my own children when I made my donation	42 (n = 68) (62%)	20 (n = 41) (49%)	62 (n = 109) (57%)
I have donated on more than one occasion	18 (n = 67) (27%)	18 (n = 45) (40%)	36 (n = 112) (32%)
I was single at the time of my donation	9 (n = 68) (13%)	20 (n = 43) (47%)	29 (n = 111) (26%)

**Table 2:** Respondents’ knowledge of use. Tabulated summary of respondents’ knowledge of the use of their donated gametes.

	Female Respondents	Male Respondents	All Respondents
I donated to someone I did not know	55 (n = 68) (81%)	37 (n = 40) (93%)	92 (n = 108) (85%)
I am aware of how many families have received my donations	49 (n = 68) (72%)	33 (n = 38) (87%)	82 (n = 106) (77%)

### Overall satisfaction

Ninety-four out of 120 female and male respondents combined, rated their donation ‘experience’. Twenty-six respondents did not answer this question. Of the 94 female and male respondents combined, 76 (48 female and 28 male) described their experience positively by rating it as either ‘good’ or ‘very good’. Table 3 highlights specific aspects of the donation process that were found to be less than satisfactory by the respondents who rated their overall donation experience positively.

**Table 3:** Unsatisfactory aspects of donation process. Aspects of the donation process that were found to be less than satisfactory by respondents who rated their overall donation experience positively.

	Female Respondents Positive Experience (n = 48)	Male Respondents Positive Experience (n = 28)	All Respondents Positive Experience (n = 76)
Clinics did not respond to queries in timely manner	2 (4%)	2 (7%)	4 (5%)
Not seen on time at clinics	4 (8%)	5 (18%)	9 (12%)
Not given support to complete Goodwill Message and Personal Description	20 (42%)	14 (50%)	34 (45%)
Not allocated a single point of contact at clinic	9 (19%)	8 (29%)	17 (22%)
Lack of information regarding expenses	10 (21%)	7 (25%)	17 (22%)
Were not confident on what information they could request regarding the donation outcome	3 (6%)	3 (11%)	6 (8%)
Unaware that additional counselling could be requested from the clinic if needed	11 (23%)	9 (32%)	20 (26%)

### Clinic communication

Five percent of respondents who rated their overall donation experience positively stated that clinic staff ‘never’ responded promptly to their enquiries, with a sperm donor writing, *‘clinics absolutely useless with any sort of communication’* (SD_47), and another male respondent concluding, *‘I got the impression that they were very busy and had more than enough donors’* (SD_43). Similarly, an egg donor described the clinic staff as very slow in contacting her after the first appointment and having *‘to contact them several times about getting an appointment’* (ED_68). Another egg donor claimed she *‘found it very hard to get through [to the clinic] in the first place—I rung and left two messages and no one called me back’* (ED_12). For one egg donor, this left her concluding, *‘I didn’t always feel that the protocols were appropriate for donors—being expected to run around and set up scans at a local clinic and find out who to speak to’* (ED_64).

Twelve percent of respondents who rated their overall donation experience positively were never seen on time when attending clinics for appointments, as a sperm donor explained,

*‘I was kept waiting too often*. *I don’t need anything from the clinic. There is no advantage to me, so why should I wait. If you want ppl [people] to donate, then don’t make it harder than it should be, especially since much can be done before they arrive’*

(SD_26).

Overall, female and male respondents frequently described clinics as busy, which led to two sperm donors feeling like a *‘burden’* (SD_47) or a *‘necessary evil’* (SD_47) to be endured by clinic staff. However, the pressures on clinic staff were commonly referred to by all respondents, and acted as justification for the poor communication they received, as the following extract from an egg donor illustrates,

*‘They’re so busy and provide excellent treatment and results*, *however the communication is poor. I was so desperate to donate that I put their communication aside’*

(ED_35).

### Staff support

Forty-five percent of respondents who rated their overall donation experience positively did not receive support to complete the Goodwill Message and Personal Description. Nine percent of respondents who rated their overall donation experience positively did not have the purpose of the Goodwill Message and Personal Description explained to them, and 13 percent were unaware of who would see the Message and Description.

Twenty-two percent of respondents who rated their overall donation experience positively reported not being allocated a single point of contact at the clinic they attended. Those male respondents who had been allocated a single point of contact, such as fertility nurses or donor co-ordinators, reported it had a positive impact on their donation experience as it acted as a source of *‘support’* (SD_45) and reduced embarrassment related to the donation as the following quote from a sperm donor illustrates,

*‘…it was always the same nurse*. *I found it a lot easier to be looked after by the same one, as we were able to get over any embarrassment issues and developed a professional relationship’*

(SD_19).

### Health information

Thirteen percent of female respondents who rated their overall donation experience positively were never given advice on contraception during or after the treatment. An egg donor wrote, *‘Nor did they [staff] tell me that my menstrual cycle would be up the wall. I’m now stressed out because when my period is late I think I may be pregnant’* (ED_25). Twenty-nine percent of female respondents who rated their overall donation experience positively stated they were never advised about the possible impact the donation could have on their own fertility, with an egg donor’s comment suggesting that she had only become aware of the possibility as a result of completing the NGDT questionnaire, *‘I was a bit unsure as to why being a donor would have a possible impact on my fertility so in that respect, [clinic] communication wasn’t clear’* (ED_35).

### Staff concern for Donor Welfare

Thirty-three percent of female respondents who rated their overall donation experience positively, reported experiencing health problems during and/or after the collection cycle, with 31 percent stating they did not receive sufficient pain relief during and after the egg collection. An egg donor wrote a graphic description of her egg collection and the *‘excruciating’* (ED_72) pain she experienced, leading to her crying and requesting to be sedated further. She described the staff as *‘showing no empathy’* (ED_72) towards her, and feeling that she was treated like a *‘piece of meat’* (ED_72), and ultimately, *‘violated’* (ED_72) after a suppository was given without notice or explanation.

Ovarian Hyperstimulation Syndrome (OHSS) was overwhelmingly reported, with two egg donors admitted to hospital, and one describing herself as treated by clinic staff as a *‘human incubator’* (ED_55). Another woman reported bed wetting for approximately two weeks after the collection. There was no correlation between those respondents who had experienced health problems, either during or after collection, and those who reported negative comments on how clinic staff treated them. Respondents suggested the donation experience could be improved by clinic staff demonstrating concern over donors’ physical well-being, even if they had donated previously, as an egg donor wrote,

*‘A bit more sympathy when you are going in on your own for a medical procedure which completely knocks you out*. *Being aware that this is frightening and confusing. I feel the clinic was a bit shoddy in explaining everything (even though I had only recently donated before…). They were also poor in explaining exactly how to inject yourself with the pen and I just had to remember from before’*

(ED_69).

Respondents wished for clinic staff’s concern to extend beyond the collection. Four respondents reported disappointment that they were not contacted by the clinic to check on their well-being, with an egg donor feeling *‘a little used as they had what the wanted (my eggs) and I didn’t feel like aftercare was a concern’* (ED_40). Another egg donor said the lack of aftercare shown towards her made her *‘feel that “you’re doing a wonderful thing” was just lip service’* (ED_17) and instead she was not *‘needed or wanted’* (ED_17). Finally, an egg donor reported being made to feel like *‘an incubator which would be used as and when it was required’* (ED_55).

### Donation facilities

Twenty-nine percent of male respondents who rated their overall donation experience positively were unsatisfied with the location of the donation rooms. Concerns were raised over the position of the donation room in relation to the rest of the clinic, i.e. the waiting room, and those who were able to see the donation room, such as fertility patients, and clinic staff. Two respondents also mentioned how despite an *‘occupied’* sign being placed on the donation room, people still tried to enter the room, which the respondents found *‘off putting’* (SD_20).

Twenty-nine percent of male respondents who rated their overall donation experience positively, were unsatisfied with the condition of the donation rooms. A respondent wrote, *‘strong soap and rough towels made washing 3 times before donation uncomfortable’* (SD_38). Another respondent referred to the hygiene of the donation room, requesting *‘a clean sheet to cover the chair’* (SD_45). One respondent had donated in an *‘ordinary toilet cubicle’* (SD_40), which he described as *‘uncomfortable’* (SD_40), as well as *‘slightly demeaning’* (SD_40).

Thirty-two percent of male respondents who rated their overall donation experience positively, stated their sample pots were not always labelled before the donation, and 36 percent of male respondents who rated their overall donation experience positively, did not feel that their donation pot was always received with discretion by the clinic staff.

### After ‘Donation’

Eight percent of respondents who rated their overall donation experience positively, were not confident on what information they could request regarding the outcome of their donation and when. Two sperm donors claimed clinic staff perceived sperm donors as *‘unemotional unconcerned automatons’* (SD_31), who were uninterested in the outcome of the donation.

Twenty-six percent of respondents who rated their overall donation experience positively, were unaware they could approach the clinic for additional counselling if required after they had donated. Twenty-two percent of respondents who rated their overall donation experience positively reported not being provided with information on the expenses they were eligible to claim for donating. Respondents described clinics as disorganised and lacking processes in place for donors, particularly after collection had taken place, such as ordering transport, having expense forms or funds readily available, providing medication and aftercare, as an egg donor explained,

*‘I had to ask for expenses rather than be offered*. *One time I had to wait for 20 minutes for a few pounds, and they knew I needed to get to work. They did not send through medication (the nasal sprays) when they said they would… They did not order me a taxi, although I asked them three times to do so for after the operation, as I was worried I would feel really ill and struggle to get home, and did not really want to stand in the street trying to find a cab… I was hassled for my payslip to prove I had a job (for the day off work payment) approximately 15 minutes before I went down for surgery, when I had asked the week before if I needed to bring anything and was told I did not’*

(ED_50).

### Donor requirements of the gamete donation process

**Fig 1:**
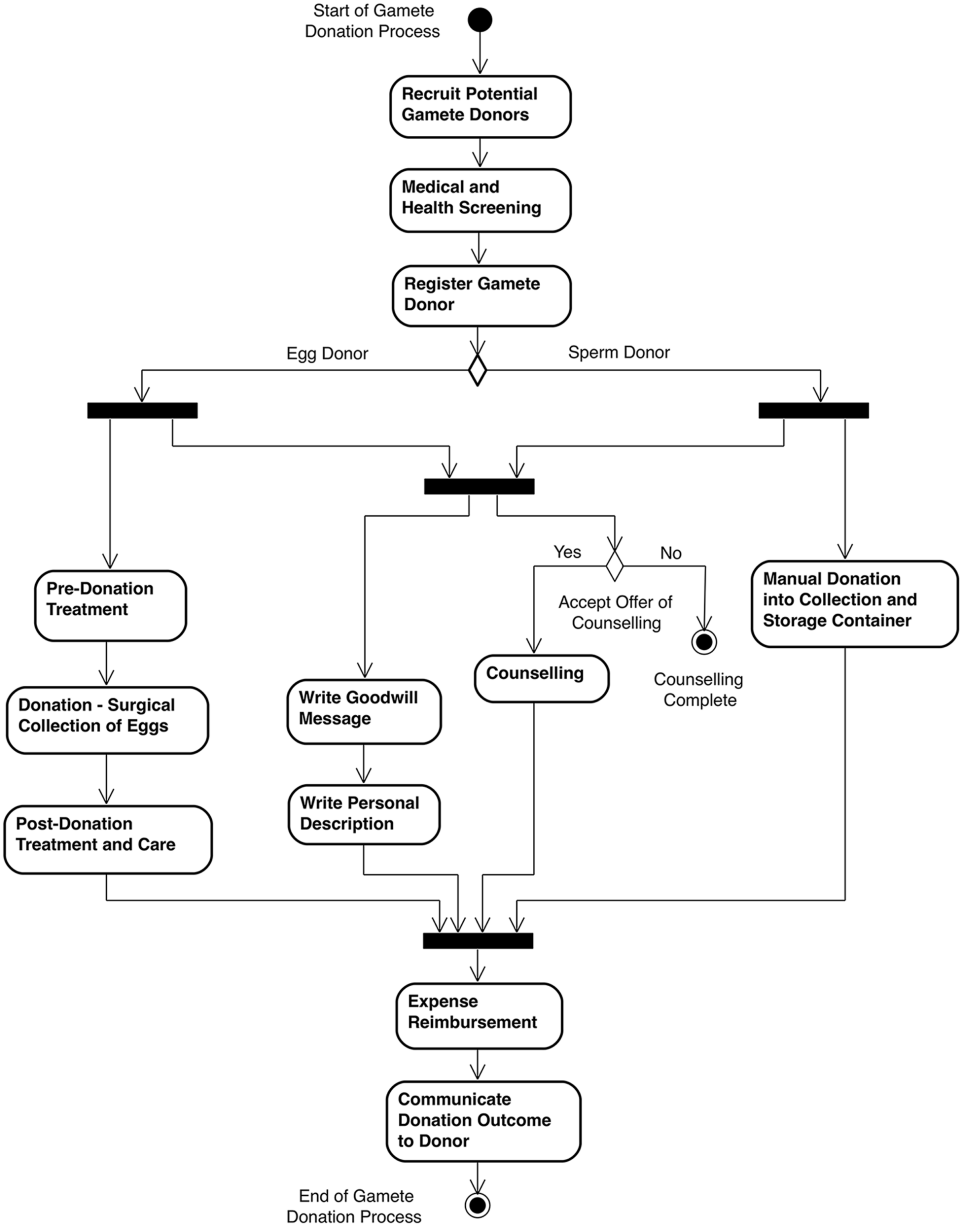
High-level activities in gamete donation process. UML Activity Diagram that depicts the high-level activities involved within the gamete donation process from the donor perspective. These activities map on to the themes of the qualitative data presented previously, namely: Clinic Communication, Staff Support, Health Information, Staff Concern for Donor Welfare, Donation Facilities, and After Donation. Each one of the activities within the diagram has the potential to negatively affect donor satisfaction of the gamete donation process if not managed effectively by the clinic staff.

Further to the qualitative analysis presented above, we have used two diagrammatic notations from the the Unified Modelling Language, namely *Activity* diagram and *Communication* diagram, along with a less formal *Expected Behaviours* diagram, to take a more holistic approach to analysis. It can be seen from the UML Activity diagram (Fig 1) that there are a considerable number of activities within the donation process where donor satisfaction can be adversely affected. These can relate to pre-donation activities, where the potential donor is recruited, undergoes medical and health screening, and formally registers; to the physical donation process itself (different activities for egg and sperm donors), access to counselling, and assistance with writing the *Goodwill Message* and *Personal Description*; along with post-donation activities, such as reimbursement of expenses and communication from the clinic at a later date about the outcome of the donation.

**Fig 2:**
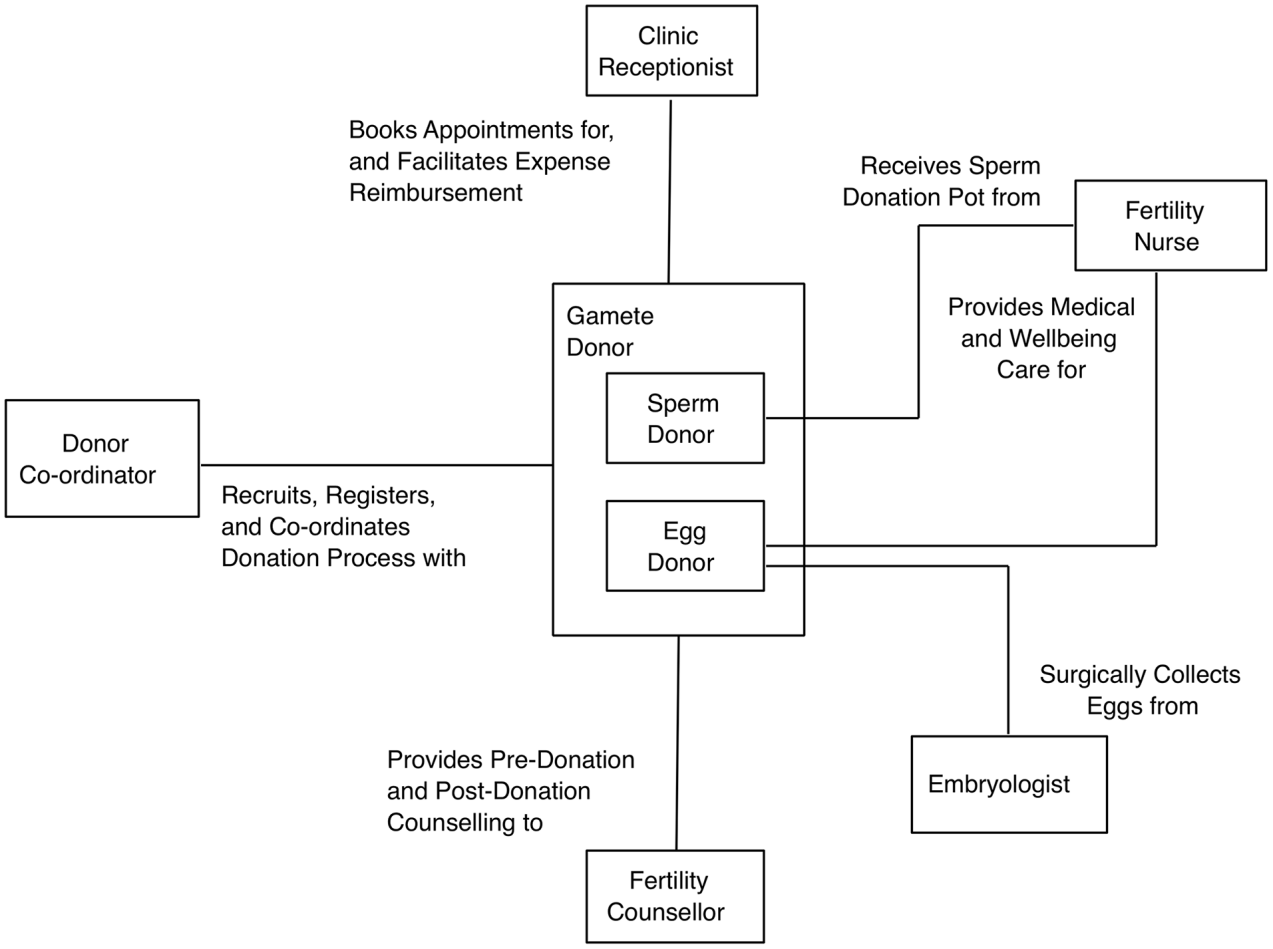
Donor requirements of relationships with clinic staff. UML Communication Diagram that depicts the requirements that donors have for their relationships with clinic staff.

The *Communication* diagram (Fig 2) conveys the minimal requirements that doonors have for their interactions and relationships with clinic staff. The potential to adversely affect donor satisfaction can occur through interactions with any one of these clinic stakeholders (Receptionist, Donor Co-ordinator, Fertility Nurse, Embryologist, or Fertility Counsellor). As such, it is important that key interactions (related to activities from Fig 1) are performed with a focus on donor well being (both emotional and physical) and their engagement with the donation process, which will lead to an increased donor satisfaction with the gamete donation process.

**Fig 3:**
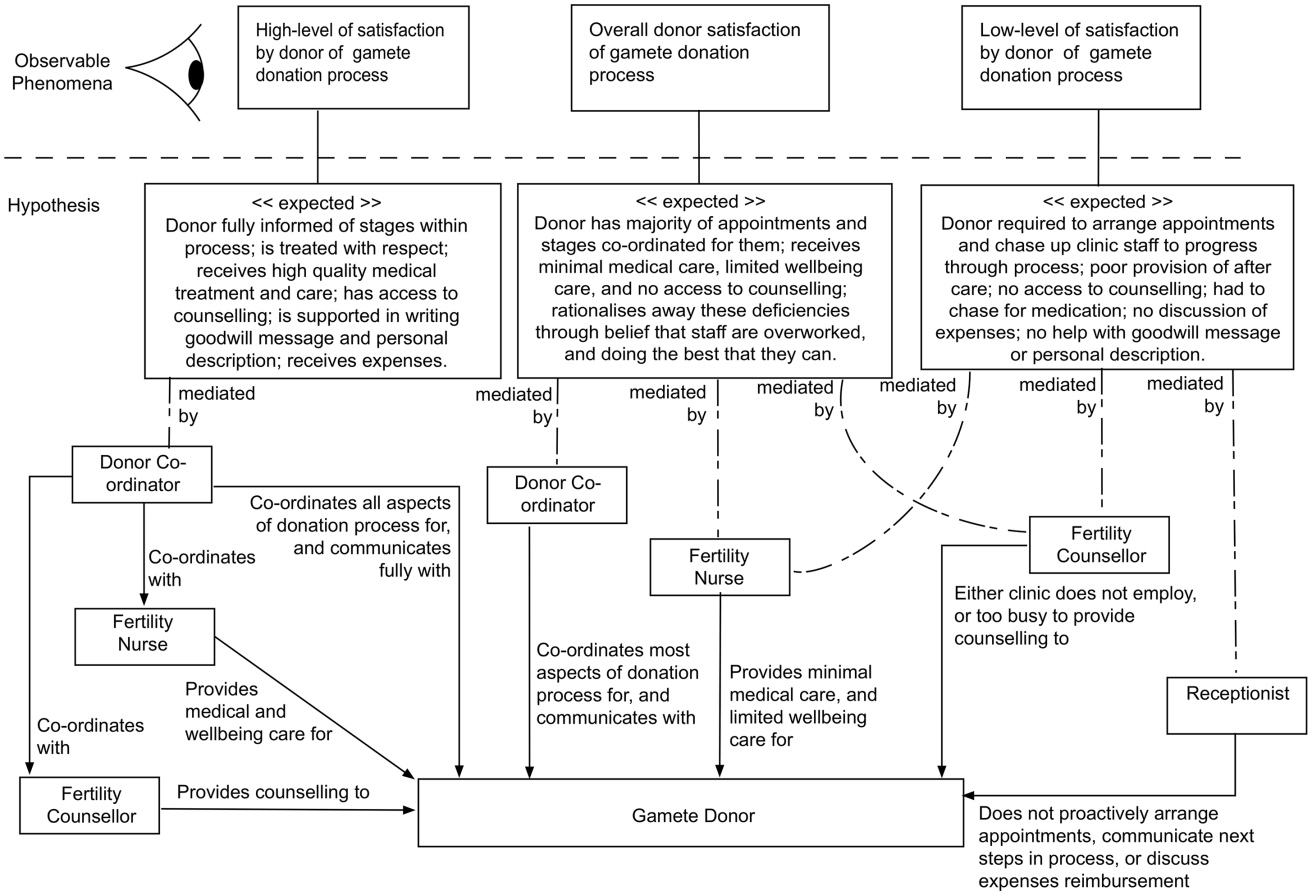
Expected behaviours diagram. Expected behaviours diagram depicting the observable phenomena of donor satisfaction; the behaviours that are hypothesised to be responsible for the donor when forumating their opinion of satisfaction; and at an abstracted level the clinic staff and the responsibilities that they carry out in relation to the gamete donor. It is hypothesised (expected) that the degree to which the donor perceives that clinic staff perform these roles and carry out the responsibilities, will affect their overall level of satisfaction with the gamete donation process.

Through linking the high-level activities in the donation process to the clinic stakeholders and the associated interactions, we are able to hypothesise reasons why donors perceive a high level of satisfaction, an overall satisfaction, or low level of satisfaction. This *rich picture* of the complex system can be conveyed using an *Expected Behaviours* diagram (Fig 3). It can be seen that the Donor Co-ordinator and Fertility Nurse are the primary clinic stakeholders who are able to affect donor satisfaction positively. Conversely, the lack of access to a Fertility Counsellor can cause a reduction in satisfaction, even if the rest of the donation process has been positive; and a poor relationship between the donor and Receptionist can also quickly reduce satisfaction, because the administrative burden associated with certain activities in the donation process (e.g. scheduling appointments, communicating next steps, expense reimbursement) can disproportionately affect a donor who is experiencing emotional and/or physical discomfort from the other activities within the wider donation process.

## Discussion

In line with previous studies [1, 13], respondents in this study lacked information on the medical aspects of the donation process (e.g. contraception, along with side effects during and/or after collection), and lacked clinic support on the administrative aspects of the donation process (e.g. writing Goodwill Message and Personal Description). Respondents in this study also wished for improved aftercare and knowledge of the outcome of their donations, as discussed elsewhere [24] (see Fig 1 for high-level activities involved in the gamete donation process). The positive reports from those respondents who had a single point of contact at the clinic, suggest it would be worthwhile implementing this practice throughout clinics in the UK. In essence, these findings add weight to the calls from other researchers for dedicated clinic staff to support gamete donors throughout the donation process [13] (see Fig 2 for relationships that donors wish they had with clinic staff).

This study also confirms the findings from other gamete donor satisfaction studies that have shown high rates of overall satisfaction, despite donors experiencing poor clinic communication [2, 42], and/or staff treatment [1] (see Fig 3). Moreover, very few respondents in this study expressed regret over their decision to donate, as other researchers have found [3, 10]. It would be easy to assume therefore that gamete donor satisfaction is not influenced by poor clinic communication, and/or staff treament, and propose that future satisfaction studies should measure different factors. The principle underlying this assumption would be that a negative experience equates to a negative satisfaction rating, but there has been scant consistent empirical evidence to support such a statement [6].

In this study, some respondents openly prioritised their motivation to donate above their need for information or assistance from clinic staff. As a result, the portrayals of gamete donors as altruistic found elsewhere [20–22] are fuelled by the implication that donors’ needs in terms of receiving information and support from clinic staff, are sacrificed in order to provide gametes for others’ fertility treatment. In essence, the donors’ aim to donate was achieved when the physical act of donation was carried out, meaning their overall satisfaction rating would barely be affected by poor clinic communication and/or staff treatment.

Yet, if gamete donor satisfaction is simply driven by donors’ motivations to donate altruistically, this does not account for the variance in donors’ ratings of certain aspects of the donation process. So, in this study, the ‘altruistic’ drive of donors did not necessarily result in identifiable ‘failings’ of a service being overlooked. Instead, donors offered low ratings for some aspects of the donation process, such as poor clinic communication. These low-rated aspects of the donation process were either justified by donors, due to perceived mitigating circumstances (e.g. staff not responding to queries in a timely manner due to them being overworked), or no excuses were offered, as seen when donors discussed whether they received eligible expenses in a timely manner. In essence, donors’ ratings reflect their beliefs about the service they should receive, and whether staff were to blame when it was lacking [5–7].

**Fig 4:**
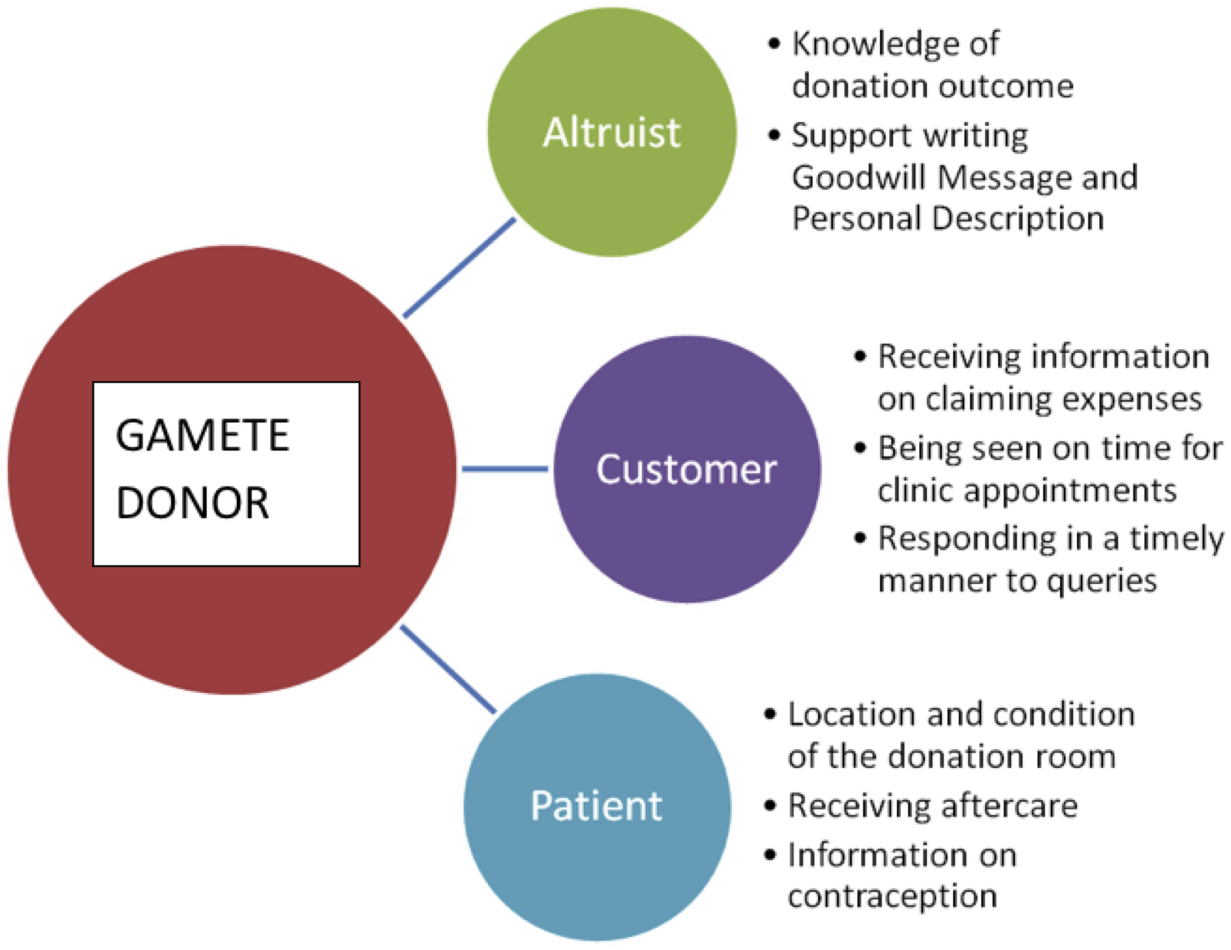
The notion of the gamete donor. The notion of the gamete donor is based around three facets, which are: gamete donor as altruist, gamete donor as customer, and gamete donor as patient. These facets are underpinned by different motivations, objectives, and requirements.

In turn, these beliefs provide insight into the notion of the ‘gamete donor’ (see Fig 4). For example, donors rated and discussed their desire for knowledge on the outcome of their donation, and their wish for support when writing the Goodwill Message and Personal Description. It can be inferred that these were expressions of donors’ concern for the infertile couple and the resulting child, thereby highlighting their altruistic motives to donate. Conversely, when donors expressed frustration at not receiving information on the expenses they could claim, or not being seen on time for clinic appointments, it suggested that donors also experienced the donation process as a ‘customer’ might when using a ‘service’. Yet, donors’ comments on aftercare, the location and condition of the donation room, and information on contraception, indicate that donors also experience the donation process as a patient might when having medical treatment. Therefore, the notion of a gamete donor appears to be made up of three facets: altruist, customer, and patient.

Deconstructing the notion of a ‘gamete donor’ in this way offers an explanation for the positive overall satisfaction reported in surveys [9], despite the negative ratings for specific aspects of the donation process. When answering questions on surveys relating to overall satisfaction and future donation, respondents answer from the position of ‘donors-as-altruists’, whereas questions on clinic communication and staff treatment are answered by respondents as ‘donors-as-customers’ or ‘donors-as-patients’. It is therefore possible to claim that clinic communication and staff treatment *are* significant factors for improving donor care. In addition, this highlights the fact that donors are able to hold more than one of these *perspectives* during their overall gamete donation journey, although we conjecture that at any particular point in time, they will be holding a single perspective for the associated phase of the donation process.

## Conclusions

Understanding the notion of a gamete donor as an altruist, customer, and patient, has policy and practice implications. Donors have a desire to feel valued from the moment they make their initial enquiry about donating, to their recovery after collection. Fertility nurses are well-placed to demonstrate care towards donors by focusing on a number of practical, everyday tasks, which patients have been found to appreciate [27] such as responding to donors’ contact in a reasonable time frame, ensuring the facilities of the donation room are reasonable, expressing concern over donors’ well being after collection, as well as taking responsibility to ensure donors’ expenses are reimbursed promptly. By introducing a single point of contact for donors, committing to a specific time frame that donors can expect to receive a response from a clinic, and making assistance available when writing the Goodwill Message and Personal Description, will help to manage donors’ expectations, as well as encourage clinics and fertility nurses to incorporate potential donors’ needs into their practices. The multifaceted notion of ‘donors’ illustrates how they can experience the donation process, which needs to be reflected in the clinic communication and staff treatment they receive.

## Supporting information

S1 Table Dataset that was used for the statistical analysis within this manuscript. (XLS)
